# Medical students’ assessment of pediatric patients - teaching and evaluation using video cases

**DOI:** 10.1186/s12909-014-0241-x

**Published:** 2014-11-13

**Authors:** Michelle Malon, Dina Cortes, Gorm Ole Greisen

**Affiliations:** Neonatal Division, Rigshospitalet, Copenhagen, Denmark; Department of Pediatrics, Hvidovre Hospital, Faculty of Health Science, University of Copenhagen, Copenhagen, Denmark; Neonatal Division, Faculty of Health Science, University of Copenhagen, Rigshospitalet, Copenhagen, Denmark; Department for Child and Adolscent Psychiatry, Region Sjaelland, Smedegade 16, Roskilde, Denmark

**Keywords:** Medical education, Video based teaching, Patient video case, Rubric score, Pediatrics

## Abstract

**Background:**

We introduced video-based teaching in pediatrics. We evaluated the impact of a pediatric video program on student performance in assessing pediatric patients presented as video cases. The program consisted of a library of pediatric videos, and inclusion of these in the teaching and examination for pediatric medicine.

**Methods:**

Medical students on a pediatric clerkship at the University of Copenhagen assessed eight short pediatric video cases during autumn 2011 and spring 2012. Two independent observers evaluated a subset of records in a pilot study. A blind evaluation was made of the written records of 37 students before, and 58 students after, the introduction of the program using a Rubric score with four domains.

**Results:**

The intraobserver interclass correlation coefficient was 0.94 and the interobserver interclass correlation was 0.71(n=25). The students’ mean total Rubric score in spring 2012 (7.0) was significantly higher (p<0.001, 95% CI 1.34–3.20) than autumn 2011 (4.7). Cohen's d was 1.1 (95% CI 0.6–1.7). Single domains scores increased significantly for general assessment (1.30 versus 0.57) (p<0.002, 95% CI 0.45–1.18), recognition of principal symptoms (1.38 versus 0.81) (p<0.008, 95% CI 0.22–0.91), appropriate diagnosis (2.28 versus 1.78) (p<0.002, 95% CI 0.16–0.82) and consistency between observed symptoms and diagnosis (1.94 versus 1.57) (p=0.0482, 95% CI 0.00–0.79).

**Conclusions:**

Students improved in evaluating pediatric patients presented as video cases after the introduction of the program. The impact on real-life situations remains to be established.

## Background

The clinical assessment of pediatric patients is difficult, and students' access to pediatric patients may also be limited because of seasonal variation and short clerkships. This is likely to limit the acquisition of competence [1]. Teaching methods that promote faster and deeper learning are often discussed among medical educators, and a supplement to ordinary teaching is needed.

Dual-code theory states that the formation of mental images aids learning [2]. Inclusion of a visual approach in teaching therefore enhances learning and the ability to recall. The theory suggests that this is partly because images and words are processed in different parts of the brain [2], and that images are coded redundantly, providing two representations rather than one [3].

Educators have explored the efficacy of using patient video cases, which, at least in theory, expose students to the complexity of actual clinical problems [4]. Although the use of video cases in medical education is not unusual, it is seldom systematic, and the literature is sparse [5]. Assessing the impact of video-based teaching is difficult and it is even harder to assess whether the skills learnt in this way can be transferred to patients in real life.

The aim of this study was to evaluate the effect of a video case teaching program in Pediatrics on students’ performances in assessing pediatric patients presented as video cases.

## Methods

Before January 2012, standard education in Pediatrics at the University of Copenhagen consisted of a 5-week pediatric clerkship. During the clerkship, students participated in daily activities at the pediatric departments as well as problem-based learning sessions and formal lectures. The clerkship ended with a 30-minute oral assessment, based on a written patient case.

In January 2012 (spring semester 2012), a video case teaching program in Pediatrics was introduced. This consisted of access to a new library of 180–200 short pediatric patient video cases on the university e-learning platform, which was available online 24/7 to medical students during their pediatric clerkship. We have previously described the content of and topics covered by the video case library [6]. According to the university log, 89% of the students (211/238) accessed the video case library during the spring semester 2012 [6], but data on the time spent watching video cases were not analyzed. Students were briefly introduced to the video case library and how to get value from it at the beginning of their clerkship. During the clerkship, but not at a specified time, students were offered a 1–2-hour video case tutorial supervised by the faculty as part of the teaching and preparation for the final assessment. A 30-second video case was included in the assessment. In total, the video-based part of the assessment was 5 minutes long, and the written patient case was shortened to 25 minutes including grading.

In a prospective study design, medical students who attended the pediatric course at the University of Copenhagen during autumn 2011 and spring 2012 semesters participated in a test, where students assessed eight video cases presenting different common conditions in pediatric patients (Table 1). Before the beginning of the autumn semester 2011, the author group, together with members of the Faculty, selected the topics of the eight video cases, to show characteristic manifestations of diseases. These video cases lasted 30–90 seconds, and each one was shown twice, before the students recorded their evaluations on pre-printed paper forms. The eight video cases were the same in both semesters and were not seen by the students in advance. In preparation for the test, students were informed that they should assess whether the child was sick or healthy and the severity of the illness. They were also asked to write down the most important findings about the video case patient to present to a senior colleague for advice (including findings about the general appearance of the child as well as the principal symptoms). Students also made at least one tentative diagnosis of the patient. The instructions for the test were also written on the pre-printed form. The test was done 1 week before the examination. Students were not allowed to discuss the video cases during the test. All test records were anonymous and stored without analysis. After the test, the video cases were discussed extensively in plenary, supervised by MM. The students provided oral feedback about their experiences and their thoughts on the video-based teaching, which were recorded by MM.

**Table 1:** **Diagnoses for the video cases**

Video case no. 1	Child of 8 months with croup
Video case no. 2	Healthy 4-month-old baby
Video case no. 3	Child of 5 years with Henoc-Schönlein vasculitis
Video case no. 4	Child of 1 year with generalized tonic-clonic seizure
Video case no. 5	4-month-old baby with severe bronchiolitis
Video case no. 6	3-month-old baby with infantile spasm
Video case no. 7	9-day-old baby with severe hypotonia, as part of trisomy 21
Video case no. 8	9-month-old child with chicken pox

To obtain as objective a measure as possible of student performances in assessing pediatric patients presented as video cases, we developed a Rubric score (Table 2), a tool that can be used to assess student competencies [1,7]. The Rubric score was constructed by the author group and consisted of four domains: general condition of the child, principal symptoms, suggested diagnoses and consistency between description, symptoms, and diagnosis. Prior to the test, the author group defined keywords for each video case in all domains, setting out the desired standard of reporting. Scoring was from 0 to 4 points in every domain. For example, in the domain concerning principal symptoms, students who assessed and described at least three correct principal symptoms in just 0–1 of the video cases were given no points, in 2–3 video cases 1 point, in 4–5 video cases 2 points and so on. The Rubric scores for each domain were summed to a total. The author group chose the domains to be similar to those used in the final assessment in Pediatrics. The standard in relation to obtainable scores was set by consensus in the author group based on general acceptance in the faculty of what could be expected from an average student. Students were at no time presented with any knowledge about the Rubric score, and it was used only for the purpose of this study, to compare student performance in assessing pediatric patients presented in the eight video cases. To evaluate the Rubric score according to distribution as well as the intra- and interobserver interclass correlation, a random group of students (n = 25) from the autumn semester 2011 was Rubric-scored twice by two independent observers (MM and GG) in a pilot study. The Rubric score was not revised after the pilot study.

**Table 2:** **Design of the Rubric score**

**Point**	**0**	**1**	**2**	**3**	**4**
**General condition of the child:** Describe the general appearance of the child with at least one observation	≤ 1 video	2–3 videos	4–5 videos	6–7 videos	All videos
**Principal symptoms:** Describe at least three correct principal symptoms	≤ 1 video	2–3 videos	4–5 videos	6–7 videos	All videos
**Suggested diagnosis:** Make correct diagnosis	≤ 1 video	2–3 videos	4–5 videos	6–7 videos	All videos
**Coherence of answers:** Consistency between the description of the general appearance of the child, the symptoms and the suggested diagnosis	≤ 1 video	2–3 videos	4–5 videos	6–7 videos	All videos
**Total**					

**Figure 1:**
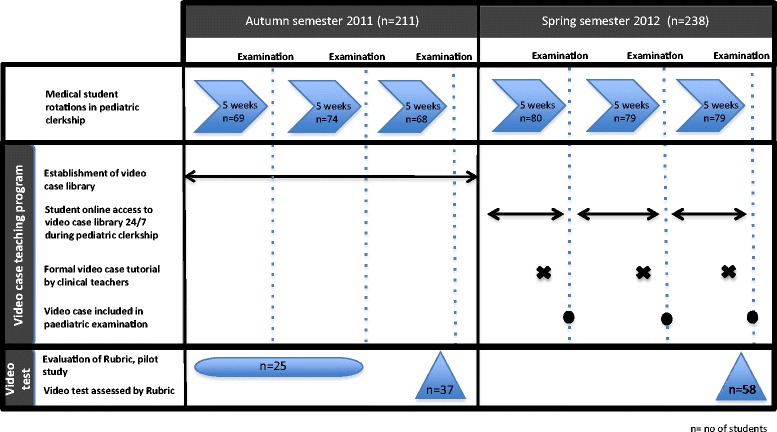
**Time line for video case teaching program and study interventions.** The study period was the autumn semester 2011 and spring semester 2012. In the first and second rotations in the autumn semester 2011, 25 students were randomly chosen for the pilot study. Students in the third rotation in the autumn semester 2011 and in the whole of the spring semester 2012 participated in the study. Using the Rubric score, records were scored blindly.

To evaluate the effect of the video case teaching program on student performance in assessing pediatric patients presented as a video case, the records of students from the third rotation in the autumn semester 2011 and spring semester 2012 were Rubric-scored blindly by MM (Figure 1). Students were matched for geographic location for attending the pediatric clerkship. Students attended the testing day were also included. Students who had not completed all eight video cases, for example because they were late, were excluded.

As described previously [6], we used the regular university system provided by the Evaluation Division of the Faculty of Health and Medical Science for student evaluation to obtain formal written feedback of the students' perceptions of video-based learning at the end of the course. This evaluation system was based on a 7-point Likert scale and supplementary qualitative comments.

Records were blinded before scoring. Data were normally distributed and analyzed using interclass correlation and a two-tailed unpaired t-test. The level of significance was set at 0.05.

## Results

A total of 25 records from the autumn semester 2011 were used in a pilot study to evaluate the intra- and interobserver interclass correlation. The intraobserver interclass correlation coefficient was 0.94 and interobserver interclass correlation was 0.71.

A total of 95 students (autumn semester 2011, n = 58 and spring semester 2012, n = 37) completed the test to evaluate the effect of the program. The groups had a comparable mean age (28.3 years in autumn 2011 and 28.5 years in spring 2012) and gender balance (36% versus 35% male in autumn and spring, respectively).

**Figure 2:**
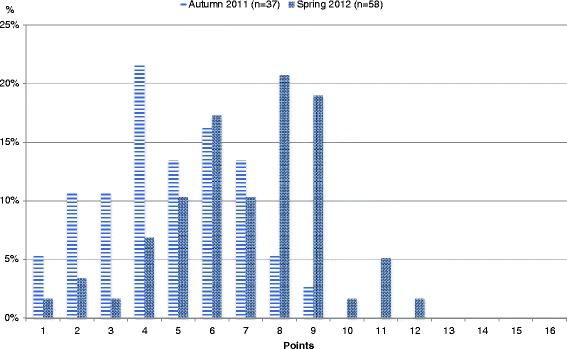
**Total Rubric score of student records before and after introduction of the video case teaching program.** The student mean total Rubric score after introduction of the video case teaching program in spring 2012 (7.0 ± 2.3) was significantly higher than the students’ total score in autumn 2011 (4.7 ± 2.0), (p < 0.001). The effect size (Cohen's d) was 1.1 (95% CI 0.6–1.7).

**Figure 3:**
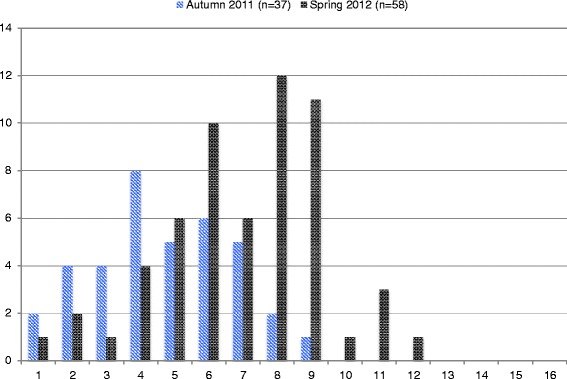
**Total Rubric score of student records before and after introduction of the video case teaching program, raw numbers.**

The student mean total Rubric score in spring 2012 was significantly higher (7.0) than the students’ total score in autumn 2011 (4.7) (95% CI 1.34–3.20, p < 0.001) (Figures 2 and 3). The effect size (Cohen's d) was 1.1 (95% CI 0.6–1.7).

After the video case program was introduced, the mean single domain Rubric scores were significantly higher for general assessment of the child (1.30 versus 0.57) (95% CI 0.45–1.18, p < 0.002), assessment of principal symptoms (1.38 versus 0.81) (95% CI 0.22–0.91, p < 0.008) and diagnoses (2.28 versus 1.78) (95% CI 0.16–0.82, p < 0.002). Improvement in consistency between description, symptoms, and diagnosis just reached the level of significance (1.94 versus 1.57) (95% CI 0.00–0.79, p = 0.0482).

As described previously [6], students reported during a plenary discussion supervised by MM that they valued the learning from the video cases. This was echoed in their written feedback to the Evaluation Division of the Faculty of Health and Medical [6]. Students in the spring semester 2012 also reported that to get maximum value, they would have appreciated more supervision and discussion about the video cases with the clinical teachers during their clerkship. Students commented positively in their feedback on the short duration of the video cases in the test as well as in the video case library.

## Discussion

In our study, students’ abilities to evaluate pediatric patients presented as video cases, as assessed by a Rubric score, significantly improved after the introduction of the video case teaching program. The data are robust, as the intra- and interobserver interclass correlation coefficients were very good, even though more independent observers would have provided further strength.

Using clinical video cases of pediatric patients in an authentic clinical context, rather than single text cases with or without still pictures, may enable students to work on developing observational skills, which otherwise develop haphazardly with clinical experience.

As the program was in several parts, our study does not give a clear picture of the causes of the observed increase in students’ Rubric scores. It is well known that students focus their attention on what will enable them to pass examinations [8-11]. This may have increased student focus on how to “handle” the patient video cases rather than on generic observational and diagnostic competence. The results of the Rubric score for consistency between the description of the general condition of the child, the specific symptoms, and the proposed diagnosis, which just reached the level of significance, may support this cynical interpretation.

Another limitation of our study is the relatively small number of students. As the composition of the Rubric will naturally influence the outcome, the choice of domains and standards for Rubric scores may also be worthy of discussion, as different choices and sets of standards might have altered the results. The Rubric score was not tested on any selection of video cases other than the eight used in this study. Our Rubric score therefore needs further evaluation to clarify its usefulness in evaluating student performance in assessing pediatric patient video cases. Supplementary focus group interviews would have provided further information about the mechanisms involved in the students’ cognitive processes when assessing the video cases.

We were surprised that many reports demonstrated little consistency, as some students described symptoms other than those the child presented, but somehow managed to make an appropriate diagnosis, or described the symptoms well but gave an entirely irrelevant diagnosis. An example from several students was the correct description of inspiratory stridor and croupal cough in video case no. 1, a child with croup, but a diagnosis of asthma or pneumonia. From the dual-code theories, the observed discrepancy may demonstrate that the students did not integrate the visual cues in the video cases with their theoretical knowledge of pediatric symptoms and illness. We speculate that this could be improved by increasing the number of supervised video tutorials, or by including more videos in the diagnosis-focused teaching sessions.

We also noted a tendency for the students to overlook and fail to report on the general appearance of the child. We speculate that this may be due to “tacit knowledge”—that students may not pay attention to, or may unconsciously assess, the general condition of the child, and focus on symptoms and diagnosis. This interpretation is supported by our unstructured observation of the apparently intuitive ability of students, even on the first days in clerkship, to identify a “normal” rather than severely ill child.

Other studies [4,12-19] that have explored video as a learning medium in medical education have reported that video-based teaching is beneficial as a supplement to ordinary clinical teaching and that confidence in recognizing abnormal findings increased [12,15]. Lee et al. [15], however, found in a study of second-year medical students given a video-enhanced case of a hypotonic infant that anxiety about starting a period in the neonatal unit did not decrease, even though the videos had been effective in teaching the students to identify abnormal findings in a newborn. We have previously shown that students’ self-reported confidence in the assessment of pediatric patients increased significantly after the introduction of video-based teaching [6].

Several studies have shown that authenticity—that is, a realistic situation and the feeling of “caring for a real patient"—is the critical feature of a video case and contributes to higher learning by enhancing case discussions [12,13]. The use of video cases also increases the meaningfulness for medical students [16]. In the test, as well as in the library, the video cases were of short duration (30–120 seconds), which students appreciated, reporting that it helped them to focus during the whole video case. De Leng et al. [16] stated that videos should not be too long, but we have not been able to find any literature describing the optimal duration of patient video cases for value maximization.

Kamin et al. [12] compared video- and text-based cases in problem-based learning in virtual and face-to-face settings. The video case group of students reported the highest confidence in their ability to recognize abnormal findings in their patients. Students also found the video cases valuable in structuring their knowledge, conceptualizing how to handle difficult situations, distinguishing abnormal from normal physical examination findings, and collaborating with their peers and their mentor to develop critical thinking.

In another study investigating the influence of video cases compared with written cases on the critical thinking process in problem-based learning, Kamin et al. [13] showed that the scores of the video case group were higher than those of the text group in all assessed parameters, except in the problem-identification stage. The authors hypothesized that students using video cases first had to perceive the information, then verbally express what they saw, before they were able to identify the problem. These students therefore had an additional step in the process. Roy and McMahon [19] conducted a cross-over study using one video-based and one text-based case and evaluated the cognitive activity for deep versus superficial thinking. They concluded that, overall, the odds of deep thinking were significantly lower using video-based cases.

Balslev et al. [20] compared the effects of a video case and a paper case on the verbal group interaction among residents and showed that the video case group yielded higher frequencies of clauses relating to data exploration, theory building and theory evaluation than the group using the paper case. They suggested that cognitive processes were stimulated by the video case, and that human working memory seemed to expand with the use of visual and auditory information. In another study, Balslev et al. [17] described shared cognition as a feature that extends beyond shared knowledge, because it includes the processes and products of clinical reasoning and builds upon individuals’ inputs in the collaborative process. They suggested that a video case is more effective than a paper case in facilitating the sharing of cognitive processes by learners engaged in team learning. They observed that many residents commented on the video case while watching, unlike the residents in the text group, who were silent during their initial reading. The authors concluded that this may have contributed to an earlier onset of causal concept links formation in the video case group. They also observed a delay in verbal interaction in the video group after the first and second playing and concluded that watching and processing the video cases may have caused cognitive overload, slowing down concept link formation. Multiple replays of the video cases, interspersed with discussion, may be beneficial. In our study, the students were not allowed to talk during the video test, because we wanted to evaluate each one individually. We were not fully aware of the impact this might have and therefore it is possible that we did not provide sufficient encouragement to students to watch and discuss the videos together. This may have lowered the effect of the use of videos and may be one reason for the rather low consistency between observations and diagnoses and the lack of a statistically significant increase during the intervention period.

The students in our study commented very positively on the use of video cases, but also expressed their need for supervision and dialogue as a critical way to achieve effective learning from the use of video cases. This is in accordance with other studies [2,4,12-14,16-18,21,22], and in line with the observations described above. De Leng et al. [16] also reported that a structured approach to watching a video case was essential.

Kamin et al. [12] investigated students’ perceptions of a virtual problem-based learning experience by comparing video cases used in virtual and face-to-face settings. Students preferred to work through the cases in face-to-face groups but agreed that the virtual experience was a worthy alternative for long-distance learning. Students from all groups reported that the cases were a good use of their time and improved their ability to solve clinical problems by giving them an opportunity to “get away from just doing and focus on learning”. However, the virtual group complained of the lack of “a barometer for how much is too much”, as they spent on average 8 to 10 hours per case, whereas the face-to-face groups spent on average 3 hours. Difficulties in prioritizing which video cases to select from the library were also an issue in our students’ feedback reports. As skill in assessing pediatric patients presented as a video case was required for the final assessment in Pediatrics, students in our program may have spent much of their self-study time using the online video case library, which may be reflected in our results.

Finally, and importantly, we have not been able to find studies that have shown a beneficial effect of patient video case-based learning on the clinical assessment of patients in real life. Further studies are needed.

## Conclusion

The availability of a formal video case teaching program was very effective in improving student ability to assess such video cases. It remains to be established if these interventions also improve their ability to assess patients in real-life situations.

### Ethics

The study was approved by the Danish Data Protection Agency, j.nr 2007-58-0015. Informed consent was obtained from all participants. All patient video cases were recorded with written informed consent from custody holders. The Danish Regional Health Research Ethics Committee was consulted, but no further approval was needed, as the study did not involve any kind of human intervention, excision of human tissue, or similar.
